# Sex-Specific Association between Social Frailty and Diet Quality, Diet Quantity, and Nutrition in Community-Dwelling Elderly

**DOI:** 10.3390/nu12092845

**Published:** 2020-09-17

**Authors:** Chi Hsien Huang, Kiwako Okada, Eiji Matsushita, Chiharu Uno, Shosuke Satake, Beatriz Arakawa Martins, Masafumi Kuzuya

**Affiliations:** 1 Department of Community Health and Geriatrics, Nagoya University Graduate School of Medicine, 65 Tsuruma-cho, Showa-ku, Nagoya 4668550, Japan; beatriz.martins@adelaide.edu.au; 2 Department of Family Medicine, E-Da Hospital, No.1, Yida Road, Jiaosu Village, Yanchao District, Kaohsiung City 82445, Taiwan; 3 School of Medicine for International Students, College of Medicine, I-Shou University, No.8, Yida Rd., Jiaosu Village, Yanchao District, Kaohsiung City 82445, Taiwan; 4 Institutes of Innovation for Future Society, Nagoya University, NIC, Chikusa Ward, Furocho, Nagoya 4648601, Japan; chiharu83724@gmail.com; 5 Graduate School of Nutritional Sciences, Nagoya University of Arts and Sciences, Takenoyama–57, Iwasakicho, Nisshin 4700196, Japan; kiwako@nuas.ac.jp (K.O.); e.matsu@nuas.ac.jp (E.M.); 6 Section of Frailty Prevention, Department of Frailty Research, Center for Gerontology and Social Science, National Center for Geriatrics and Gerontology, 7–430 Morioka-cho, Obu City 4748511, Japan; satakes@ncgg.go.jp; 7 Department of Geriatric Medicine, Hospital, National Center for Geriatrics and Gerontology, 7–430 Morioka-cho, Obu City 4748511, Japan; 8 Adelaide Geriatrics Training and Research with Aged Care (G-TRAC Centre), Discipline of Medicine, Adelaide Medical School, University of Adelaide, 61 Silkes Rd, Paradise, Adelaide 5075, Australia; 9 National Health and Medical Research Council Centre of Research Excellence in Frailty and Healthy Ageing, University of Adelaide, Adelaide 5005, Australia

**Keywords:** social frailty, diet quantity, diet quality, nutritional status, sex difference

## Abstract

The effects of social frailty on diet and nutrition are under-investigated. Our study aimed to assess the association between social frailty and diet quality, diet quantity, and nutrition over a 3-year period in community-dwelling older Japanese adults. This prospective cohort study recruited individuals aged ≥60 years from a community college and followed up 666 participants annually. Social frailty was determined using a 4-item questionnaire. Diet quantity (energy and macronutrient intake) and diet quality (dietary diversity score and Diet Quality Index-International) were assessed using a food frequency questionnaire. Nutrition was evaluated using the Mini-Nutritional Assessment (MNA). Out of the 666 participants (56.5% women), 250 (37.5%) were categorized as having social prefrailty or frailty. Regarding diet quantity, energy intake (β = −1.59kcal/kg/day, *p* < 0.01) and nutrient intake (protein intake, β = −0.08g/kg/day; fat intake, β = −0.06g/kg/day; carbohydrate intake, β = −0.18g/kg/day; fiber intake, β = −0.01g/kg/day; all *p* < 0.05) were lower in men with social prefrailty or frailty than in men with social robustness. Dietary diversity score (β = −0.25, *p* = 0.01) and MNA score (β = −0.32, *p* = 0.04) decreased in men with social prefrailty or frailty. However, these associations were not observed in women. Social frailty is associated with lower dietary intake, poor diet quality, and poor nutrition among community-dwelling older men. Future studies are required to determine the benefits of sex-specific interventions targeting social frailty on nutritional outcomes.

## 1. Introduction

Frailty has several multifactorial etiologies involving an individual’s physical, psychological, and social domains [1]. Although physical frailty has been widely investigated, the definition of social frailty and its impacts on health outcome have been relatively insufficiently investigated [2]. Recently, several indices measuring social frailty have been developed and validated based on the following four critical domains in the conceptual model of social frailty: general resources, social behavior/activities, social resources, and fulfillment of social needs [3]. Additionally, social frailty in older adults has been reported to be associated with poor physical function, disability, cognitive impairment, depression, and higher mortality [4,5,6,7,8]. The social vulnerability index established according to the deficit accumulation model was associated with physical frailty, disability, and survival [9,10,11]. A theory-guided approach also demonstrated the independent association of social frailty with unfavorable health outcomes, including malnutrition [12]. However, other associated health adverse effects of social frailty are not well understood yet, such as its effects on dietary intake, including dietary quantity and quality.

Nutrition plays significant roles in not only leading to but also mediating and reversing physical frailty in older adults [13]. Malnourished older adults are more vulnerable to becoming physically frail than well-nourished older adults, and multifaceted interventions including nutritional approaches are recommended for physical frailty management [1,14]. A meta-analysis found that nutritional status might become worse in older adults without spouses, with difficulty walking or climbing stairs, and with prior records of hospitalization [15]. Additionally, social inequality including socioeconomic deprivation, subjective loneliness, poor social support, and inaccessible social resources might be associated with malnutrition in individuals with low social status [16,17,18]. Nevertheless, these studies mainly assess nutrition using malnutrition screening tools with insufficient personal dietary assessment.

Dietary habits, which comprise diet quality and quantity, were associated with physical frailty and metabolic syndromes [19,20]. A meta-analysis demonstrated that dietary patterns characterized with high fruit, vegetable, and whole grain intake may be associated with reduced risk of frailty [19]. Additionally, one prospective cohort study reported that diet quality characterized by lower score of the Diet Quality Index-International (DQI-I) was associated with incident physical frailty [21]. Diet diversity, another indicator of diet quality, was shown to be associated with physical frailty and social support. On the other hand, the association between economic disadvantage with an income threshold and dietary variety was noted in men, but not in women [22]. Another study found that social support and self-preparation of meals could affect the dietary diversity score (DDS) in women, but not in men [23]. Additionally, social isolation and subjective loneliness seemed to be associated with significant increases in malnutrition risk among older people [17]. Therefore, additional longitudinal studies are required to confirm the impact of socioeconomic status, such as social isolation and social frailty, on diet quality, diet quantity, and nutrition in older adults according to sex. Hence, the present study aimed to assess the sex-specific association between social frailty and diet quality, diet quantity, and nutritional status during a 3-year period in community-dwelling older Japanese adults.

## 2. Materials and Methods

### 2.1. Study Design and Participants

We designed a 3-year prospective cohort study, entitled Nagoya Longitudinal Study for Healthy Elderly, to investigate the trajectory of nutrition in the community-dwelling older adults. Older adults aged between 60 and 89 years were recruited from a community college in Nagoya City, Japan, from 2014 to 2017. Individuals with the following characteristics were excluded from the study: older adults who were unable to perform the basic activities of daily living (e.g., feeding, shower, dressing, toileting, transferring, and maintaining continence) and to walk independently. The present study was conducted in accordance with the guidelines of the Declaration of Helsinki and approved by the ethics committee of the Nagoya University Graduate School of Medicine (approval number 2013–0055–2) and the ethics committee of the Nagoya University of Arts and Sciences (approval number 83, approved on 10 September 2013). All participants provided written consent for inclusion in the study. Other cross-sectional and longitudinal results have been published elsewhere [24,25,26].

### 2.2. Definition of Social Frailty

Social frailty was assessed using a 4-item questionnaire derived from a social frailty screening tool that has been validated to correctly stratify the risk of death and disability in community-dwelling Japanese older adults [5]. Our questionnaire comprised the assessment of general resources, social resources, social behavior, and fulfillment of basic social needs based on Bunt’s social frailty concept, and inquired about financial difficulty (need support vs. no need for support), household status (living alone vs. not living alone), social activity (non-participation in social activities vs. regular participation in social activities), and influential contact with neighbors (total scores of the Lubben Social Network Scale–6 <12 points vs. ≥12 points) [3]. A deficit score of 2 or higher was graded as social frailty, 1 as prefrailty, and 0 as robustness [5].

### 2.3. Measures

Participants’ baseline characteristics, including age, sex, educational level, socioeconomic status, body height and weight, medical history, and medication use, were obtained through in-person interviews. The Charlson Comorbidity Index (CCI) was constructed to investigate disease burden [27]. Depressive symptoms were assessed using the Geriatric Depression Scale (GDS) [28]. The primary outcome measures were nutritional status, diet quality, and diet quantity. The Mini-Nutritional Assessment (MNA) tool was used to assess nutritional status [29]. Diet quality and diet quantity were measured using a semi-quantitative food frequency questionnaire (FFQ), which was validated comparable with weighed dietary records for 7 continuous days [30].

### 2.4. Dietary Assessment

A 34-item FFQ adapted from a validated FFQ included 29 pre-specified food groups, and 10 culinary methods were used to investigate dietary intake [31]. The food groups were as follows: noodles, breads, cooked rice, potatoes, fish and meat, soybeans and soybean products, light-colored vegetables, mushrooms, algae, green and yellow vegetables, nuts and seeds, boiled dishes, vinegared dishes, marinated dishes, fruits, salty foods (pickles, salted cod roe, kelp simmered in sweetened soy sauce, salted fish, and salted plums), soup dishes (potage, consommé, clear soup, and miso soup), seasonings (Worcester sauces, soy sauces), confectioneries, fats and oils, sugar (including honey and jam), and alcoholic beverages. Dietary habits were traced back over the previous 1–2 months, including every meal in 1 week. The frequencies of food consumption and the portions of food consumed were recorded self-reportedly and further confirmed by trained dietitians and research staffs. An in-person interview was conducted to check all inputs for blanks, errors, and mismatches, lasting about 10–15 min for each participant. Portion size was quantified using pictographs of individual food items, which helped to estimate the standardized portion size (e.g., one serving of rice is one small bowl cooked with an approximate weight of 150 g). We used a standardized software program (“Mr. Excel Nutrition”) to convert the standardized portion sizes of the foods into nutrients, including estimating multiple nutrients of one food automatically [30]. All foods consumed by the subjects were available in the Standard Tables of Food Composition in Japan 2015 (7th revised edition) and nutrient intake (including alcohol intake) was calculated accordingly [32]. Portion sizes on mixed dishes were based on our experience or ingredient and nutrient facts for convenience food.

### 2.5. Diet Quality and Diet Quantity

Diet quality was evaluated using the DDS and DQI-I. The DDS was determined by the amount of food groups consumed daily (fish and shellfish, meat, eggs, milk, soybean/soybean products, green and yellow vegetables, potatoes, seaweeds, fruits, and fats and oils), and previous studies demonstrated that the adherence to these 10 food groups was associated with increase in high-level functional capability and decrease in frailty severity for community dwelling elderly [33,34]. Eating more than 3 times a week for each food group was assigned a score of 1 with a maximal total sum score of 10, indicating the best dietary diversity [33].

The DQI-I was generated based on the following four main components: variety (overall food group variety and within-group variety for protein source), adequacy (vegetable group, fruit group, grain group, fiber, protein, iron, calcium, and vitamin C), moderation (total fat, saturated fat, cholesterol, sodium, and empty calorie foods), and overall balance of food intake (macronutrient ratio and fatty acid ratio) [35]. Details of the scoring system have been described elsewhere [35]. The total score ranged from 0 to 94, with a higher score indicating a better diet quality.

Diet quantity was presented as the average daily nutrient intake and macronutrient composition (percentages of total calories intake) based on the Standard Tables of Food Composition in Japan (seventh revised edition) and The Food and Nutrition Board of the Institutes of Medicine (IOM) [36,37]. Total energy intake (including alcohol and fiber intake) and nutrient intake was adjusted by ideal body weight (height^2^ (m^2^) × 22) [38].

### 2.6. Statistical Analyses

Differences in participants’ baseline characteristics with or without social prefrailty and frailty were based on an independent *t*-test or Kruskal–Wallis test for continuous variables or a chi-squared test for categorical variables, as appropriate. The longitudinal association between social frailty status and dietary intake, DDS, and MNA score over a 3-year period was analyzed using a generalized estimating equation adjusting for sociodemographic variables (age and educational level) and health-related covariates (body mass index [BMI], GDS, and CCI scores) [39]. Living status was not adjusted as it was already included in the definition of social frailty. Regarding managing missing data, multiple imputation with standard fully conditional specification was utilized to create five imputed datasets [40]. Pooled results of estimates of interest, according to Rubin’s rules, are presented in the Supplementary Materials [41]. All tests for significance were two-sided at the 95% level (*p* < 0.05). All statistical analyses were performed using the International Business Machines Corporation (IBM) Statistical Package for the Social Sciences Statistics for Windows, version 25.0 (IBM Corp., Armonk, NY, USA).

## 3. Results

### 3.1. Participants’ Characteristics

**Figure 1:**
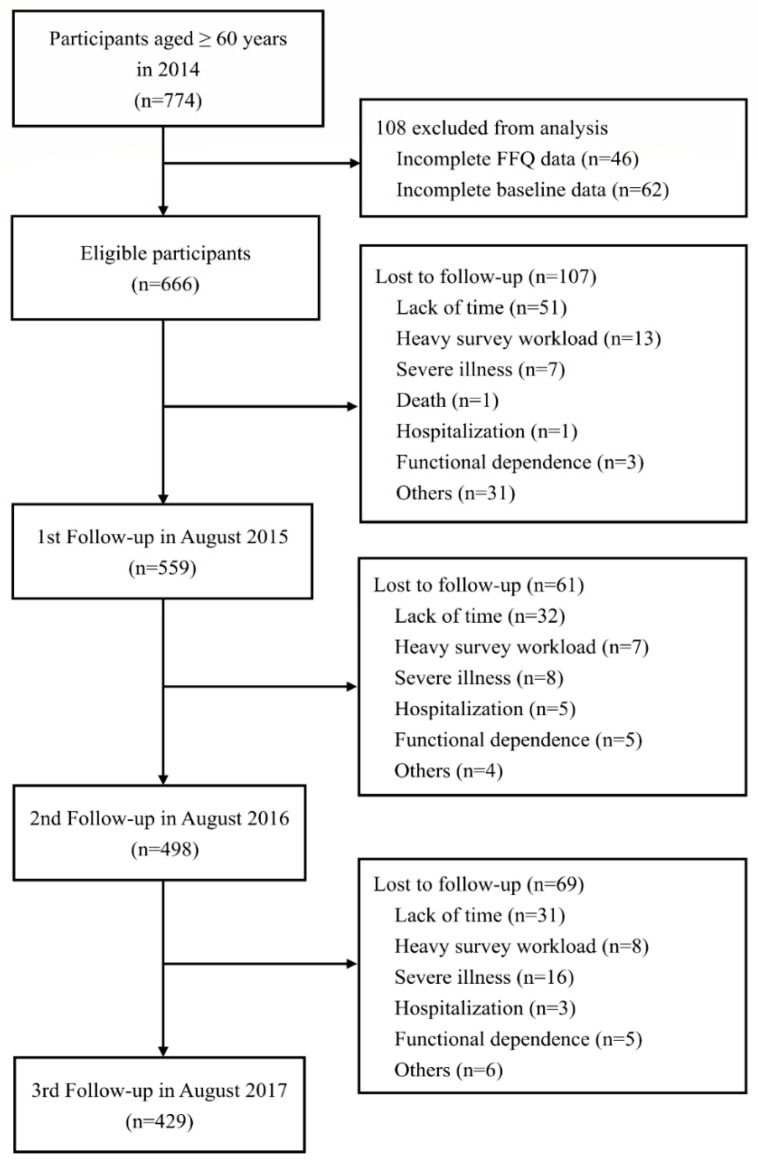
Flowchart of study participants and follow-up.

A total of 774 participants were included at baseline. We excluded 108 participants with more than 10% missing values regarding dietary information or incomplete baseline characteristics from the analysis. In total, 666 participants (56.5% women) were assessed annually between 2014 and 2017. During the 3-year follow-up period, 237 enrollees dropped out primarily owing to lack of time, as reported by themselves. Figure 1 shows the flowchart of the recruitment process, dropout reasons, and the number of dropouts. Comparing the baseline characteristics of dropouts and non-dropouts, no significant differences were observed in all variables except the DQI-I (Table S1). 

The recruited participants had a mean age of 69.4 years (standard deviation: 4.4 years) at baseline. Older adults aged greater than 65 years comprised 89.3% of all participants. A total of 250 (37.5%) participants were categorized as having social prefrailty or frailty. The MNA scores were 26.6 ± 2.1 and 25.7 ± 2.4 in men and women, respectively. The CCI scores were 3.4 ± 1.3 and 3.0 ± 1.0 in men and women, respectively. The total daily energy intakes were 32.6 ± 8.0 and 36.4 ± 8.3 kcal/kg in men and women, respectively. The total daily protein intakes were 1.1 ± 0.3 and 1.4 ± 0.4 g/kg in men and women, respectively. The macronutrient ratios for carbohydrate, fat, and protein were 57.8 ± 5.6%, 53.9 ± 5.1%, and 28.3 ± 4.4% in men; 31.0 ± 4.1%, 13.9 ± 1.9%, and 15.1 ± 1.9% in women. Male participants with social prefrailty and frailty had lower MNA scores, lower DDS, and a lower level of dietary intake in all components. However, these differences were not observed in women (Table 1).

### 3.2. Effects of Social Frailty on Diet Quality and Diet Quantity

In men, energy intake (β = −1.59, *p* < 0.01) and nutrient intake (protein intake, β = −0.08; fat intake, β = −0.06; carbohydrate intake, β = −0.18; fiber intake, β = −0.01; all *p* < 0.05) were lower in the social prefrailty and frailty groups than in the social robustness group, but the macronutrient composition was similar in both groups (Table 2). On the contrary, social frailty status was not associated with energy intake, nutrient intake, and macronutrient composition in women.

Regarding diet quality, the DDS was associated with age, CCI score, and social frailty status in men and with age and GDS and CCI scores in women (Table 3). Notably, only men with social prefrailty and frailty had a decreased DDS (β = −0.25, *p* = 0.01), but this finding was not observed in women. The DQI-I was not associated with social frailty status in either men or women (Table 3). After replacing the missing values using the multiple imputation method, our findings remained essentially unchanged (Tables S2 and S3).

The MNA score was associated with BMI and GDS and CCI scores in both men and women. However, men with social prefrailty and frailty had a lower MNA score (β = −0.32, *p* = *0*.04) than in men without social prefrailty and frailty. The MNA score difference was also observed in women; however, it was not statistically significant (Table 4). In the model without adjusting for BMI, social prefrailty and frailty still showed a negative effect on MNA score in men (β = −0.39, *p* = *0*.02). The results remained consistent regardless of the replacement of missing data (Table S3).

## 4. Discussion

Our findings suggest that social frailty is associated with diet quantity, diet quality, and nutritional status in community-dwelling older men, but not in older women. The sex difference in response to social frailty might trigger future research and investigation to investigate sex-specific interventions.

The gender differences in diet quantity consumed in the elderly were less investigated. In the present study, although energy intake per kilogram of ideal body weight was lower in men than in women at baseline, men still had a significantly higher total energy and macronutrient intake than women. This discrepancy is due to the larger portion of muscle mass in men than in women, as there is a lower metabolic rate in body composition for men [42]. However, the proportion of macronutrient composition was similar between men and women in our results.

Social interactions had gender-specific effects on diet quality, diet quantity, and nutritional status in the present study. Among quantitative studies, a multi-country survey in Europe found that social isolation was associated with having less than three meals a day and less fruit or vegetable consumption without gender differences [43]. Another large cross-sectional survey showed that older adults who ate alone regardless of sex had significantly lower daily consumption of green or yellow vegetables, fruits, and fats/oils in Japan than those who ate together with other individuals [44]. However, low DDS was more associated with men than women who ate alone in this study [44]. Our consistent findings corroborated that social frailty had a larger negative influence on dietary habits and nutritional status for men who might not usually prepare meals for themselves. The loss of food diversity manifested by DDS and subsequent malnutrition occurred in men with social prefrailty or frailty; however, the DQI-I remained unchanged. The discrepancy might be attributed to the fact that lower DQI-I scores in non-dropouts at baseline potentially underestimated the negative consequences of social frailty on DQI-I. In addition, energy intake played a role in affecting DDS and DQI-I scores, albeit to a lesser extent for the latter, especially if energy came from saturated fats and cholesterol, leading to obesity and health adverse events [45,46]. However, to facilitate nutritional screening and assessment in clinical practice, DDS, which is a less time-consuming, self-reported, feasible and reliable measure with a positive correlation with protein and micronutrient intake, was chosen to evaluate the diet quality in our study [47]. This tool without the requirement of large amounts of dietary recall information could find the difference among men with and without social prefrailty/frailty. In contrast, women, who are usually more competent at cooking and preparing meals, had the ability to maintain adequate nutrient intake even if they were socially frail [48]. Therefore, social frailty is a risk factor for inadequate nutrient intake and compromised diet diversity and malnutrition among older men, but not among older women.

Unsurprisingly, age, disease burden, and depression were associated with diet quality and diet quantity, consistent with the findings of a previous meta-analysis [15]. Moreover, increased BMI played a potential protective role against malnutrition [49]. Therefore, treatment strategies for social frailty should incorporate disease control, depression screening and management, and nutritional promotion in addition to offering social resources and enhancing social interactions.

A variety of interventions targeting social isolation and loneliness have been developed, including individual interventions, group interventions, and mixed interventions [50]. A systematic review suggested that information communication technology, internet-based interventions, and community-engaged programs with productive engagement might be beneficial in increasing social networks and decreasing loneliness among older individuals [50,51]. One national survey in Canada demonstrated that eating out might increase the likelihood of co-eating with someone else [52], in which the behavior could further increase social interaction. Therefore, educating older adults with social frailty to use social networking services (e.g., social media and online communities) and dine out more often could potentially not only ameliorate social frailty but also improve dietary intake and nutrition, especially for men.

The main strength of this study is the use of a prospective cohort design, subsequently confirming the longitudinal association between social frailty status and diet and nutrition. Nevertheless, this study has several limitations. First, considering that the participants who were willing to participate in our study were relatively more socially active than those who were reluctant, our findings might not be generalizable to homebound older adults who are “severely” socially isolated. Although these older adults are hardly approached for investigation, lower diet quality, diet quantity, and nutritional status are more expectedly observed in older adults who are “severely” socially isolated than in older adults who are socially active. Second, the self-administered FFQ might result in recall bias and classification error, particularly in older adults with social frailty. Moreover, concomitant cognitive impairment may have influenced the reporting of dietary intake. To minimize the gap in data collection, the survey was assisted by food pictographs and additionally confirmed by dieticians. Third, dropouts during follow-up possibly compromise the robustness of the inference. However, sensitivity analysis using the multiple imputation method produced consistent evidence. Lastly, as a result of cultural differences, our diagnostic criteria of social frailty, which are sensitive to older Japanese adults, should be modified depending on living environment and transportation convenience in other countries. Nevertheless, proactive comprehensive intervention strategies for social frailty should be recommended and implemented as early as midlife.

## 5. Conclusions

The present study reveals that social frailty is associated with lower dietary intake, poor diet quality, and poor nutritional status among older Japanese men, but not among women. Sex-specific approaches targeting social frailty on nutritional outcomes are required to determine the benefits of interventions.

## Figures and Tables

**Table 1:** Participants’ baseline characteristics by sex and social frailty status.

	Men			Women		
Variable ^†^	Social Robustness(N = 194)	Social Prefrailty and Frailty (N = 97)	*p* Value *	Social Robustness(N = 222)	Social Prefrailty and Frailty (N = 153)	*p* Value *
Age, years	69.9 (4.3)	69.5 (4.5)	0.45	68.7 (4.5)	69.9 (4.4)	0.01
Educational level, N (%)						
≤9 years	8 (4.2)	6 (6.3)	0.18	11 (5.0)	13 (8.7)	0.11
10–12 years	63 (33)	40 (42.1)		113 (51.6)	86 (57.3)	
>12 years	120 (62.8)	49 (51.6)		95 (43.4)	51 (34.0)	
Economic status, N (%)						
Need support	0 (0)	2 (2.1)	<0.01	0 (0)	9 (6.0)	<0.01
Self-supporting	157 (82.8)	88 (92.6)		175 (79.9)	112 (74.7)	
Well off	34 (17.8)	5 (5.3)		44 (20.1)	29 (19.3)	
BMI, kg/m^2^	23.2 (2.6)	23.3 (2.3)	0.95	22.1 (2.6)	21.7 (2.7)	0.07
MNA, scores	26.8 (2.1)	26.2 (2.1)	0.04	26.0 (2.3)	25.2 (2.3)	<0.01
Dietary diversity score ^§^	1.7 (1.4)	1.3 (1.3)	0.01	1.8 (1.2)	1.9 (1.3)	0.71
DQI-I	47.9 (10.2)	47.7 (9.1)	0.87	47.5 (9.5)	47.9 (9.0)	0.70
CCI, scores	3.5 (1.4)	3.4 (1.2)	0.74	2.9 (1.0)	3.2 (1.1)	0.02
Polypharmacy (≥5 medications), N (%)	30 (15.7)	20 (20.1)	0.31	15 (6.8)	15 (10.0)	0.24
Dietary intake						
Energy intake, kcal/day	2015.3 (476.7)	1854.3 (402.0)	0.01	1862.4 (409.9)	1867.2 (403.9)	0.91
Energy intake, kcal/kg/day ^‡^	33.5 (8.2)	31.0 (7.3)	0.01	36.3 (8.3)	36.7 (8.5)	0.67
Carbohydrate intake, g/kg/day ^‡^	4.3 (1.1)	4.0 (1.0)	0.04	4.7 (1.1)	4.7 (1.1)	0.79
Fat intake, g/kg/day ^‡^	1.1 (0.4)	1.0 (0.3)	0.03	1.3 (0.4)	1.3 (0.4)	0.61
Protein intake, g/kg/day ^‡^	1.2 (0.3)	1.1 (0.3)	0.01	1.4 (0.4)	1.4 (0.4)	0.62
Fiber intake, g/kg/day ^‡^	0.2 (0.1)	0.2 (0.1)	0.01	0.3 (0.1)	0.3 (0.1)	0.93
Retinol activity equivalent, μg/day	284.5 (77.4)	268.4 (84)	0.01	336.6 (77.7)	340.4 (86.8)	0.42
Vitamin D, μg/day	4.2 (1.5)	3.9 (1.6)	0.04	4.7 (1.7)	4.6 (1.7)	0.45
Tocopherol, mg/day	4.1 (0.6)	3.9 (0.7)	0.01	4.5 (0.6)	4.4 (0.7)	0.04
Vitamin K, μg/day	103.4 (32.2)	95.7 (33.9)	<0.01	123.3 (33.8)	121.1 (34.7)	0.25
Vitamin B1, mg/day	0.5 (0.1)	0.5 (0.1)	0.02	0.5 (0.1)	0.5 (0.1)	0.21
Vitamin B2, mg/day	0.6 (0.1)	0.6 (0.1)	0.78	0.6 (0.1)	0.6 (0.1)	<0.01
Niacin, mg/day	7.8 (1.6)	7.4 (1.7)	<0.01	8.1 (1.6)	7.9 (1.7)	0.09
Vitamin B6, mg/day	0.6 (0.1)	0.6 (0.1)	<0.01	0.6 (0.1)	0.6 (0.1)	0.05
Vitamin B12, mg/day	3.8 (1.2)	3.6 (1.3)	0.07	4.2 (1.3)	4.1 (1.3)	0.39
Folate, μg/day	143 (35.1)	133.6 (33.8)	<0.01	164.1 (37.7)	161.2 (37.3)	0.18
Vitamin C, mg/day	48.9 (17.1)	43.2 (16.7)	<0.01	60.1 (18.1)	58.3 (17.7)	0.08
Sodium, mg/day	3859.6 (1324.7)	3372.2 (1129.9)	<0.01	3940.7 (1280.3)	3742.1 (1238.3)	0.14
Potassium, mg/day	2383.5 (746.6)	2074.7 (686.8)	<0.01	2453.2 (697.4)	2432.8 (674.0)	0.78
Calcium, mg/day	619.6 (221.9)	555.9 (184.6)	0.02	632.1 (186.6)	655.5 (195.4)	0.25
Magnesium, mg/day	255.5 (75.7)	225.1 (65.2)	<0.01	251.8 (69.7)	251.3 (67.0)	0.94
Phosphate, mg/day	1078.1 (298.6)	960.3 (257.5)	<0.01	1070.0 (281.6)	1085.7 (269.1)	0.59
Iron, mg/day	7.8 (2.7)	6.9 (2.1)	0.01	7.8 (2.2)	7.9 (2.3)	0.93
Zinc, mg/day	8.1 (2.1)	7.2 (1.8)	<0.01	8.0 (2.1)	8.1 (1.9)	0.82
Copper, mg/day	1.1 (0.3)	1.0 (0.3)	<0.01	1.1 (0.3)	1.1 (0.3)	0.75
Manganese, mg/day	2.6 (0.6)	2.3 (0.6)	<0.01	2.5 (0.6)	2.4 (0.6)	0.71
Macronutrient composition						
Carbohydrate intake, % total energy	57.5 (5.4)	58.3 (6.1)	0.27	54 (4.7)	53.7 (5.6)	0.62
Fat intake, % total energy	28.5 (4.2)	28.0 (4.9)	0.41	30.9 (3.8)	31.0 (4.6)	0.82
Protein intake, % total energy	14.0 (1.9)	13.7 (2.0)	0.18	15.0 (1.9)	15.2 (1.9)	0.37

Abbreviations: BMI, body mass index; MNA, Mini-Nutritional Assessment; CCI, Charlson Comorbidity Index; DQI-I, Diet Quality Index-International. * Significant differences (*p* < 0.05) using chi-squared test of independence for categorical variables and independent *t*-test or Kruskal–Wallis test for continuous variables. ^†^ All values are mean (standard deviation) unless specified. ^‡^ Adjusted for ideal body weight (height^2^ (m^2^) × 22). ^§^ Ranged from 0 to 10 points, a larger value indicates higher diet diversity.

**Table 2:** Longitudinal nutrient intake changes in men and women with social prefrailty or frailty compared to the socially robust group ^†.^

	Men				Women			
Variable	GEE β Estimates *	*p* Value	95% CI		GEE β Estimates *	*p* Value	95% CI	
			Lower Limit	Upper Limit			Lower Limit	Upper Limit
Energy intake, kcal/kg/day ^‡^	−1.59	<0.01	−2.49	−0.69	0.41	0.47	−0.68	1.49
Protein intake, g/kg/day ^‡^	−0.08	<0.01	−0.12	−0.04	0.03	0.15	−0.01	0.08
Fat intake, g/kg/day ^‡^	−0.06	0.01	−0.10	−0.02	0.03	0.26	−0.02	0.08
Carbohydrate intake, g/kg/day ^‡^	−0.18	0.01	−0.31	−0.05	0.01	0.89	−0.14	0.16
Fiber intake, g/kg/day ^‡^	−0.01	0.01	−0.02	−0.003	<0.01	0.82	−0.01	0.01
Macronutrient composition								
Protein intake, % total energy	−0.17	0.32	−0.50	0.16	0.20	0.15	−0.07	0.46
Fat intake, % total energy	−0.12	0.74	−0.80	0.57	0.31	0.28	−0.26	0.88
Carbohydrate intake, % total energy	0.27	0.56	−0.65	1.19	−0.52	0.15	−1.22	0.18

Note: * GEE β estimates reflect the mean changes in the dependent variable for every 1-year increase in time. ^†^ Model was adjusted for age, body mass index, educational level, Geriatric Depression Scale score, and Charlson Comorbidity Index score. ^‡^ Adjusted for ideal body weight (height^2^ (m^2^) × 22).

**Table 3:** Factors influencing longitudinal changes based on the dietary diversity score and the Diet Quality Index-International by sex.

	Men				Women			
Variable	GEE β Estimates *	*p* Value	95% CI		GEE β Estimates *	*p* Value	95% CI	
			Lower Limit	Upper Limit			Lower Limit	Upper Limit
**a. Factors influencing longitudinal changes based on the dietary diversity score by sex**								
Age, years	**0.04**	**0.01**	**0.01**	**0.07**	**0.03**	**0.02**	<0.01	0.06
BMI, kg/m^2^	−0.01	0.81	−0.05	0.04	−0.03	0.16	−0.07	0.01
Educational level								
≤9 years	0.00				0.00			
10–12 years	−0.36	0.23	−0.95	0.23	0.11	0.54	−0.25	0.47
>12 years	−0.10	0.73	−0.70	0.49	0.25	0.21	−0.14	0.64
GDS	−0.01	0.70	−0.05	0.04	**−0.03**	**0.04**	−0.07	−0.002
CCI	**0.09**	**0.02**	**0.02**	**0.16**	**0.08**	**0.03**	0.01	0.16
Social frailty status								
Social robustness	**0.00**				0.00			
Social prefrailty and frailty	**−0.25**	**0.01**	**−0.44**	**−0.05**	0.08	0.41	−0.11	0.27
**b. Factors influencing longitudinal change based on the Diet Quality Index-International by sex**								
Age, years	0.07	0.60	−0.18	0.31	0.09	0.37	−0.10	0.28
BMI, kg/m2	−0.11	0.56	−0.50	0.27	−0.12	0.44	−0.42	0.18
Educational level								
≤9 years	0.00				0.00			
10–12 years	2.15	0.24	−1.41	5.70	0.29	0.84	−2.54	3.12
>12 years	0.28	0.88	−3.44	3.99	−1.18	0.43	−4.11	1.75
GDS score	−0.02	0.92	−0.36	0.33	0.10	0.49	−0.18	0.38
CCI score	−0.14	0.53	−0.58	0.30	−0.31	0.25	−0.83	0.21
Social frailty status								
Social robustness	0.00				0.00			
Social prefrailty and frailty	−0.26	0.71	−1.64	1.12	−0.08	0.90	−1.35	1.19

Note: Bold values denote statistical significance at the *p* value < 0.05 level. Abbreviations: BMI, body mass index; GDS, Geriatric Depression Scale; CCI, Charlson Comorbidity Index. * GEE β estimates reflect the mean changes in the dependent variable for every 1 year increase in time.

**Table 4:** Factors influencing longitudinal change based on the Mini-Nutritional Assessment score by sex.

	Men				Women			
Variable	GEE β Estimates *	*p* Value	95% CI		GEE β Estimates *	*p* Value	95% CI	
			Lower Limit	Upper Limit			Lower Limit	Upper Limit
Age, years	0.01	0.73	−0.03	0.04	0.00	0.86	−0.03	0.04
BMI, kg/m^2^	0.38	<0.01	0.31	0.45	0.47	<0.01	0.41	0.53
Educational level								
≤9 years	0.00				0.00			
10–12 years	−0.50	0.18	−1.23	0.22	0.06	0.84	−0.58	0.70
>12 years	−0.03	0.94	−0.74	0.68	0.22	0.51	−0.43	0.86
GDS score	−0.16	<0.01	−0.23	−0.09	−0.22	<0.01	−0.27	−0.16
CCI score	−0.22	<0.01	−0.30	−0.14	−0.25	<0.01	−0.37	−0.14
Social frailty status								
Social robustness	0.00				0.00			
Social prefrailty and frailty	−0.32	0.04	−0.62	−0.02	−0.23	0.12	−0.51	0.06

Note: Bold values denote statistical significance at the *p* value < 0.05 level. Abbreviations: BMI, body mass index; GDS, Geriatric Depression Scale; CCI, Charlson Comorbidity Index. * GEE β estimates reflect the mean changes in the dependent variable for every 1 year increase in time.

## Supplementary Materials

The following are available online at https://www.mdpi.com/2072-6643/12/9/2845/s1, Table S1. Baseline characteristics of non-dropouts and dropouts, Table S2. Longitudinal association between social frailty status and nutrient intake and composition after missing value replacement, Table S3. Longitudinal association between social frailty status and dietary diversity score and Mini-Nutritional Assessment score after missing value replacement.
