# Supplementary material for: Sex-Specific Association between Social Frailty and Diet Quality, Diet Quantity, and Nutrition in Community-Dwelling Elderly
Source: Nutrients. 2020 Sep 17;12(9):2845. doi: 10.3390/nu12092845 (PMC7551288; doi:10.3390/nu12092845)
Supplement: Supplementary file 1 [file nutrients-12-02845-s001.zip › 20200820 appendix.docx]

Table S1. Baseline characteristics of non-dropouts and dropouts

| Variable^†^ | Non-dropouts (N=429) | Dropouts (N=237) | *P* value^*^ |
| --- | --- | --- | --- |
| Age, years | 69.2 (4.3) | 69.7 (4.6) | 0.20 |
| Sex, female, N (%) | 220 (57.1) | 156 (55.5) | 0.68 |
| Educational level, N (%) |  |  |  |
| ≤9 years | 16 (4.2) | 23 (8.2) | 0.08 |
| 10–12 years | 179 (46.5) | 129 (46.1) |  |
| >12 years | 190 (49.4) | 128 (45.7) |  |
| Economic status, N (%) |  |  |  |
| Need support | 7 (1.8) | 5 (1.8) | 0.90 |
| Self-supporting | 311 (80.8) | 230 (82.1) |  |
| Well off | 67 (17.4) | 45 (16.1) |  |
| BMI, kg/m^2^ | 22.5 (2.6) | 22.6 (2.9) | 0.41 |
| MNA, scores | 26.1 (2.3) | 26.1 (2.3) | 0.87 |
| Dietary diversity score^§^ | 1.7 (1.3) | 1.8 (1.4) | 0.49 |
| DQI-I | 44.0 (7.3) | 53.5 (9.8) | <0.01 |
| CCI, scores | 3.2 (1.2) | 3.3 (1.2) | 0.39 |
| Polypharmacy (≥5 medications), N (%) | 44 (16.5) | 37 (19.9) | 0.36 |
| Dietary intake |  |  |  |
| Energy intake, kcal/kg/day^‡^ | 34.6 (8.0) | 35.0 (8.9) | 0.53 |
| Carbohydrate intake, g/kg/day^‡^ | 4.4 (1.1) | 4.6 (1.1) | 0.05 |
| Fat intake, g/kg/day^‡^ | 1.2 (0.4) | 1.2 (0.4) | 0.30 |
| Protein intake, g/kg/day^‡^ | 1.3 (0.4) | 1.3 (0.4) | 0.36 |
| Fiber intake, g/kg/day^‡^ | 0.3 (0.1) | 0.3 (0.1) | 0.96 |
| Mineral intake, g/kg/day^‡\|\|^ | 0.3 (0.1) | 0.3 (0.1) | 0.29 |
| Retinol activity equivalent,  μg/1000 kcal/day | 213.8 (69.0) | 219.7 (75.2) | 0.30 |
| Vitamin D, μg/1000 kcal/day | 8.3 (3.9) | 8.8 (4.0) | 0.16 |
| Tocopherol, mg/1000 kcal/day | 8.1 (2.5) | 8.2 (2.4) | 0.50 |
| Vitamin K, μg/1000 kcal/day | 215.5 (83.6) | 213.3 (77.3) | 0.74 |
| Vitamin B1, mg/1000 kcal/day | 0.9 (0.3) | 1.0 (0.3) | 0.75 |
| Vitamin B2, mg/1000 kcal/day | 1.1 (0.3) | 1.2 (0.3) | 0.29 |
| Niacin, mg/1000 kcal/day | 15.0 (5.0) | 15.3 (5.1) | 0.50 |
| Vitamin B6, mg/1000 kcal/day | 1.1 (0.4) | 1.2 (0.3) | 0.67 |
| Vitamin B12, mg/1000 kcal/day | 7.5 (3.2) | 7.8 (3.3) | 0.25 |
| Folate, μg/1000 kcal/day | 290.9 (95.8) | 289.0 (90.4)) | 0.80 |
| Vitamin C, mg/1000 kcal/day | 103.8 (43.9) | 101.5 (41.4) | 0.50 |
| Macronutrient composition |  |  |  |
| Carbohydrate intake, % total  energy | 55.8 (5.5) | 55.4 (5.8) | 0.38 |
| Fat intake, % total energy | 29.7 (4.3) | 30 (4.7) | 0.35 |
| Protein intake, % total energy | 14.5 (2.0) | 14.6 (2.0) | 0.52 |

Abbreviations: BMI, body mass index; MNA, Mini-Nutritional Assessment; CCI, Charlson Comorbidity Index; DQI-I, Diet Quality Index-International

^*^Significant differences (*P*<0.05) using chi-squared test of independence for categorical variables and independent t-test or Kruskal–Wallis test for continuous variables

^†^All values are mean (standard deviation) unless specified

^‡^Adjusted for ideal body weight (height^2^ [m^2^] × 22)

^§^Ranged from 0–10 points, a larger value indicated higher diet diversity

^||^Mineral includes sodium, potassium, calcium, magnesium, phosphorus, iron, zinc, copper, and manganese

Table S2. Longitudinal association between social frailty status^*^ and nutrient intake and composition after missing value replacement^§^

|  | Men | | | |  | Women | | | |
| --- | --- | --- | --- | --- | --- | --- | --- | --- | --- |
| Variable | B^†^ | *P* value | 95% CI | |  | B^†^ | *P* value | 95% CI | |
|  |  |  | Lower limit | Upper limit |  |  |  | Lower limit | Upper limit |
| Energy intake, kcal/kg/day^‡^ | -1.63 | <0.01 | -2.54 | -0.72 |  | 0.28 | 0.62 | -0.83 | 1.38 |
| Protein intake, g/kg/day^‡^ | -0.08 | <0.01 | -0.10 | -0.06 |  | 0.03 | 0.25 | -0.02 | 0.08 |
| Fat intake, g/kg/day^‡^ | -0.06 | 0.01 | -0.10 | -0.02 |  | 0.02 | 0.34 | -0.03 | 0.07 |
| Carbohydrate intake, g/kg/day^‡^ | -0.18 | 0.01 | -0.32 | -0.05 |  | -0.01 | 0.94 | -0.16 | 0.15 |
| Mineral intake, g/kg/day^‡^ | -0.02 | <0.01 | -0.03 | -0.02 |  | -0.01 | 0.41 | -0.02 | 0.01 |
| Fiber intake, g/kg/day^‡^ | -0.01 | 0.01 | -0.02 | -0.01 |  | <0.01 | 0.79 | -0.01 | 0.01 |
| Macronutrient composition |  |  |  |  |  |  |  |  |  |
| Protein intake, % total energy | -0.16 | 0.33 | -0.49 | 0.16 |  | 0.19 | 0.17 | -0.08 | 0.45 |
| Fat intake, % total energy | -0.10 | 0.77 | -0.73 | 0.52 |  | 0.30 | 0.29 | -0.26 | 0.87 |
| Carbohydrate intake, % total energy | 0.25 | 0.59 | -0.22 | 0.72 |  | -0.50 | 0.17 | -1.20 | 0.21 |

^*^Results were presented as the social prefrailty and frailty group compared to the social robustness group as reference

^†^Adjusted for age, body mass index, educational level, Geriatric Depression Scale score, and Charlson Comorbidity Index score

^‡^Adjusted for ideal body weight (height^2^ [m2] × 22)

^§^Missing values were replaced using multiple imputation method

Table S3. Longitudinal association between social frailty status and dietary diversity score and Mini-Nutritional Assessment score after missing value replacement^§^

|  | Men | | | |  | Women | | | |
| --- | --- | --- | --- | --- | --- | --- | --- | --- | --- |
| Variable | B^†^ | *P* value | 95% CI | |  | B^†^ | *P* value | 95% CI | |
|  |  |  | Lower limit | Upper limit |  |  |  | Lower limit | Upper limit |
| Dietary diversity score |  |  |  |  |  |  |  |  |  |
| Social robustness | 0.00 |  |  |  |  | 0.00 |  |  |  |
| Social prefrailty and frailty | -0.25 | 0.01 | -0.34 | -0.15 |  | 0.08 | 0.44 | -0.11 | 0.27 |
| Mini-Nutritional Assessment score |  |  |  |  |  |  |  |  |  |
| Social robustness | 0.00 |  |  |  |  | 0.00 |  |  |  |
| Social prefrailty and frailty | -0.35 | 0.03 | -0.65 | -0.04 |  | -0.24 | 0.10 | -0.53 | 0.04 |

^†^Adjusted for age, body mass index, educational level, Geriatric Depression Scale score, and Charlson Comorbidity Index score

^§^Missing values were replaced using the multiple imputation method
